# Identifying Desired Features That Would Be Acceptable and Helpful in a Wrist-Worn Biosensor–Based Alcohol Intervention: Interview Study Among Adults Who Drink Heavily

**DOI:** 10.2196/38713

**Published:** 2023-02-02

**Authors:** Veronica L Richards, Saahas Rajendran, Robert L Cook, Robert F Leeman, Yan Wang, Cindy Prins, Christa Cook

**Affiliations:** 1 Department of Epidemiology University of Florida Gainesville, FL United States; 2 Edna Bennett Pierce Prevention Research Center The Pennsylvania State University University Park, PA United States; 3 Department of Health Education and Behavior University of Florida Gainesville, FL United States; 4 Department of Health Sciences Bouvé College of Health Sciences Northeastern University Boston, MA United States; 5 College of Nursing University of Central Florida Orlando, FL United States

**Keywords:** transdermal, biosensor, alcohol, intervention, qualitative interview, patient preferences, mobile phone

## Abstract

**Background:**

Alcohol misuse is highly prevalent in the United States and results in a huge financial and public health burden. Current alcohol reduction treatments are underused, and there is a critical need for innovation in the field. Transdermal alcohol biosensors measure alcohol use passively and continuously and may be helpful tools in alcohol interventions. To date, however, alcohol biosensors have not been widely used to directly intervene on alcohol use. There is a new wrist-worn biosensor that could be used to help people reduce their drinking, although it is unclear how best to incorporate such a device into an alcohol intervention.

**Objective:**

We aimed to identify desired features that would be acceptable and helpful in a wrist-worn biosensor–based alcohol intervention for adults who drink heavily.

**Methods:**

Participants were recruited through an alcohol contingency management study, a contact registry, and participant referral. To qualify, participants had to be aged at least 40 years, report drinking at least twice per week, and indicate interest in reducing their drinking. We conducted a semistructured interview with each participant via Zoom (Zoom Video Communications, Inc). The interview guide addressed general thoughts on the wrist-worn biosensor, how participants thought a wrist-worn biosensor could be used to help people quit or reduce drinking, types of information that participants would want to receive from the biosensor, how they would want to receive this information, and how they thought this information could be used to change their behavior. Interviews were transcribed verbatim and analyzed using thematic analysis.

**Results:**

The sample comprised 20 adults (mean age 55.1, SD 6.1 years; 11/20, 55%, women; and 17/20, 85%, Black). Of the 20 participants, 9 (45%) had previous experience with the Secure Continuous Remote Alcohol Monitor continuous alcohol monitoring ankle biosensor from participating in an alcohol contingency management study. The desirable features could be grouped into 5 main themes: features that would influence willingness to use the biosensor (it should look attractive and be both comfortable to wear and accessible), personalized messaging (personalized biosensor-based prompts and feedback could be helpful), preference for time wearing the biosensor (for some, just wearing the biosensor could have an intervention effect), sharing data with others (this was appealing to many but not to all), and mental health support (many felt that mental health support could be incorporated into the biosensor).

**Conclusions:**

Five main themes that would maximize interest in using a wrist-worn biosensor for alcohol intervention were identified. Taken together, the identified themes could inform the development of a just-in-time adaptive intervention that uses a wrist-worn biosensor to help adults who drink heavily reduce their alcohol use.

## Introduction

### Background

Alcohol use in the United States is highly prevalent, with almost 70% of adults reporting past-year use and >50% reporting past-month use [1]. Similarly, patterns of potentially harmful drinking are common among adults in the United States, with >25% reporting past-month binge drinking (a pattern of drinking that is estimated to bring the blood alcohol concentration level to ≥0.08 g/dL; ≥5 and ≥4 drinks per day for men and women, respectively) and approximately 6% reporting past-month heavy drinking (binge drinking on ≥5 days in the past month or consuming >7 and >14 drinks per week for women and men, respectively) [1,2]. In 2010, alcohol misuse (ie, drinking in a way that could be harmful to the individual or those around them) cost the United States US $249 billion in expenses related to health care (11% of the total alcohol-related cost), law enforcement (10%), and motor vehicle crashes (10%), as well as in lost workplace productivity (72%), three-quarters of which was related to binge drinking [3,4]. Alcohol misuse is responsible for >95,000 deaths per year in the United States, or 261 deaths each day [5].

Although approximately 14.5 million persons in the United States were estimated to have an alcohol use disorder (AUD) in 2019, only approximately 7% received any treatment [1]. Furthermore, <4% of the people with an AUD were prescribed any Food and Drug Administration–approved medication to treat their disorder [6]. The limited number of persons seeking treatment for AUD and the high prevalence of alcohol misuse indicate the need for innovation in alcohol interventions. Current treatment options may be limited by high costs, inconvenient hours, delays in access, and social stigma [7-10]. Technology-based interventions can potentially help to overcome these challenges [11]. To date, internet- and mobile phone–based interventions have been explored with favorable outcomes [11-14] but still require high participant effort (eg, the individual must frequently report their alcohol use and thus should be highly motivated to participate).

Transdermal alcohol biosensors can passively and continuously measure the approximately 1% of ethanol that is excreted through the skin by measuring transdermal alcohol concentration (TAC) [15] and may be an effective tool to help individuals reduce their alcohol use. Evidence from other fields supports the use of wearable technology as a tool for meaningful behavior change in adults because mobile biosensors can provide insights into a person’s health and help them to understand how their lifestyle may be affecting aspects of their health [16]. The notion that biobehavioral feedback—facilitated by, for example, a mobile sensor—can provide motivation to alter behaviors is supported by several behavior change theories, including social cognitive theory, in which self-efficacy plays an important role in behavior change [17-19]. Receiving feedback on physiological processes allows individuals to observe tangible changes in their health that would usually go unobserved but precede more obvious changes (eg, change in heart rate preceding weight loss) [16]. This feedback can lead to increased self-efficacy and increased motivation for behavior change because specific behaviors can more saliently be associated with physiological change [16].

Biosensor-based feedback has successfully been used in the context of smoking and smoking cessation; for example, Papathanasiou et al [20] used mobile heart rate sensors to provide insights into how smoking is associated with higher resting heart rates (a risk for cardiovascular disease). Furthermore, a smoking cessation intervention that leveraged portable carbon monoxide sensors reported promising results, with significant shifts in attitudes toward quitting (eg, readiness to quit and confidence in quitting) [21]. Longer-term (ie, 3 months after the intervention) results revealed increased quit rates and continuous abstinence [22]. This intervention built off several studies that suggested that receiving real-time biometric data from carbon monoxide sensors could be educational and motivational, thus leading to positive changes related to smoking attitudes and behaviors [23-26]. Evidence for the effectiveness of biofeedback also exists in the realm of food consumption and obesity [27]; for example, Teufel et al [28] demonstrated that biofeedback via electrodermal activity was associated with improved food-related self-efficacy and perceived stress.

Until recently, most research using transdermal alcohol biosensors was conducted using the Secure Continuous Remote Alcohol Monitor (SCRAM; Alcohol Monitoring Systems) continuous alcohol monitoring (CAM) device. Although this device has been well validated [29-31], it is quite large, locked on the ankle, and often used in the criminal justice system [32]; thus, it is associated with a social stigma that serves as a barrier to its use in future alcohol interventions [33].

### Objectives

Newer, wrist-worn biosensors are being developed, and these offer great promise for future alcohol interventions. An example of a commercially available wrist-worn biosensor is the BACtrack Skyn (BACtrack, Inc), which is lighter, less expensive, and collects data more frequently (every 20 seconds compared with every 30 minutes) than the SCRAM CAM anklet [29]. However, because wrist-worn alcohol biosensors have not been widely used for formal alcohol interventions, and they currently do not provide any information back to the user, it is unclear how best to incorporate such a device into an alcohol intervention. To better understand how a wrist-worn biosensor could be used in a future alcohol intervention, we sought to gather perspectives from individuals with, as well as those without, previous experience with the ankle-based SCRAM CAM alcohol biosensor. The main aim of this study was to identify desired features that would be acceptable and helpful in a wrist-worn biosensor–based alcohol intervention for adults who drink heavily.

## Methods

### Qualitative Interviews

We used in-depth qualitative interviewing methods in this study. In-depth interviews are especially useful when discussing sensitive topics (eg, alcohol misuse) that persons may be uncomfortable speaking about in a group setting [34]. Qualitative interviews allow researchers to gather stakeholder input and can be used to inform intervention design [35]. Qualitative interviews also allow researchers to explore greater context and meaning compared with quantitative approaches.

### Participants

Participants were 20 adults recruited from an alcohol contingency management (CM) study (n=9, 45%), a contact registry (n=9, 45%), and participant referral (n=2, 10%). To participate, individuals had to (1) be aged at least 40 years (criterion for the CM study), (2) report drinking at least twice per week and indicate interest in reducing their drinking, and (3) be able to read and speak English. The age criterion was kept consistent among all recruitment sources because of possible differences in the use of technology between young adults and middle-aged and older adults. Participants from the CM study all had experience with the SCRAM CAM ankle biosensor. The sample size was driven by a saturation approach (ie, when no new information was being found) [36]. All participants provided informed consent before participation and were compensated US $25.

### Procedures

Interviews were conducted using Zoom (Zoom Video Communications, Inc), a secure, cloud-based videoconferencing service [37]. All interviews were completed remotely owing to the COVID-19 pandemic and conducted by 1 researcher (VR). After obtaining consent and before beginning the interview, the interviewer obtained permission to record each session. The interviews lasted approximately 1 hour.

We developed a semistructured interview guide (available upon request) that included questions regarding the features of a potential wrist-worn biosensor–based intervention. After the interviewer shared a photograph of the Skyn wrist-worn biosensor and described its features to the participants, they were asked for their general thoughts on the device. Participants were asked how they thought a wrist-worn biosensor could be used to help people quit or reduce drinking and to think about how it could help them, specifically. They were also asked about the types of information they would want to receive from the biosensor, how they would want to receive this information, and how they thought this information could be used to change their behavior. In addition, participants were asked about their current alcohol use and previous attempts to quit or reduce their drinking.

Two researchers (VR and SR) met weekly to review the interviews (for details, refer to the *Data Analysis* section) and assess the data for saturation. The insights from this process also helped to inform and adapt questions asked in future interviews. During the final 4 interviews, preliminary findings were shared with the participants to discern whether there were other areas we had not identified and to enhance rigor in the findings.

### Data Analysis

We used Zoom to transcribe each digital interview verbatim. After initial transcription, the transcripts were checked for accuracy. We used the 6-phase approach to thematic analysis described by Braun and Clarke [38], which follows the deductive methodology of searching for categories in empirical data. A thematic approach was used because we sought to identify patterns within the responses that may not adhere to a particular theoretical framework [38]. After familiarizing ourselves with the data, 2 researchers (VR and SR) independently generated initial codes from the data using descriptive coding [39], meeting weekly to discuss coding for each transcript until 100% agreement was reached. Although it is not a part of traditional thematic analysis, we engaged in constant comparison after each interview to ensure the development of any emergent categories that were not covered in the initial codebook [36]. We identified potential overarching themes across the initially identified codes. We assessed the data within each theme to determine whether coherent patterns were formed and reassessed any data that did not fit within the identified themes, recoding as needed. All authors provided feedback on the initial coding scheme, and codes were adjusted to incorporate this feedback. We then finalized and described each theme. Finally, representative quotations were selected for presentation and included in this manuscript. All analyses were conducted using NVivo (version 12; QSR International) [40].

To ensure rigor, we followed the evaluative criteria formulated by Lincoln and Guba [41]. Peer debriefing was conducted on a biweekly basis, as part of mentee-mentor discussions (VR and CC). At each biweekly meeting, we discussed recent interviews, codes, and emergent themes. The 2 primary coders maintained an audit trail, including notes from interviews, postinterview summaries, and annotations made during the coding process to examine whether personal thoughts may have influenced study findings.

### Ethics Approval

All procedures were reviewed and approved by the institutional review board of the University of Florida (IRB202003223).

## Results

### Overview

The sample comprised 20 adults (mean age 55.1, SD 6.1 years; 11/20, 55%, women; and 17/20, 85%, Black). Of the 20 participants, 9 (45%) had previous experience with the SCRAM CAM ankle biosensor from participating in an alcohol CM study. Of these 9 participants, 1 (11%) also participated in a pilot study in which they wore the wrist-worn biosensor for a week. All 9 participants who completed the CM study had a history of heavy drinking, but only 5 (56%) reported current alcohol use; current severity of drinking was not assessed. Of the 11 participants who had not completed the CM study, all reported current heavy alcohol use; 1 (9%) participant had previously received treatment for alcohol use.

The desired features that would maximize interest in using a wrist-worn biosensor for alcohol intervention were grouped into five themes: (1) features that would influence willingness to use the biosensor, (2) personalized messaging, (3) preference for time wearing the biosensor, (4) sharing data with others, and (5) mental health support. A description of each theme is provided in Textbox 1. Each theme represented several codes, discussed in the following sections; for example, personalized messaging included the codes *real-time alerts* and *summary data*.

**Themes identified as desired features of a wrist-worn biosensor–based alcohol intervention that would be acceptable and helpful to adults who drink heavily.**

- Themes and descriptions
  Features that would influence the willingness to use the biosensor
    Participant’s thoughts about general features that would affect biosensor uptake (eg, how it looks, comfort while wearing it, and accessibility)
  Personalized messaging
    Individualized feedback from the biosensor about the participant’s alcohol use
  Preference for time wearing the biosensor
    Preferences related to time spent wearing the biosensor in alignment with the participant’s drinking goals (eg, quit drinking vs reduce drinking)
  Sharing data with others
    Preferences related to sharing the participant’s drinking information with their friends, family members, or provider
  Mental health support
    How mental health support could be incorporated into an intervention using the biosensor

### Features That Would Influence the Willingness to Use the Biosensor

The theme *features that would influence willingness to use the biosensor* refers to participants’ thoughts about general features that would affect biosensor uptake. Codes in this theme included participant sentiment regarding a wrist-worn biosensor, preference for a wrist-worn biosensor, how it looks, and comfort while wearing it. Participants indicated that they wanted a biosensor that was comfortable to wear and looked attractive. Those who had previous experience with the SCRAM CAM ankle biosensor overwhelmingly indicated (7/9, 78%) that they would have preferred wearing the wrist-worn biosensor. Participants commented that the wrist-worn device looked more comfortable and more discreet than the SCRAM CAM ankle biosensor, and thus they would prefer it:

I think this [Skyn] is better than the anklet [SCRAM CAM]...this is much better, and it looks like something that I would wear as like a piece of jewelry, that’s to me that’s what it looks like....To me, this would be more comfortable to wear.

Woman, aged 59 years

The issue of accessibility was also brought up, and a suggestion was made for a “borrowing system”:

My real concern about a program that would use tech is just that it be made accessible to folks...a borrowing system where the devices are owned by care providers and they’re kind of leased out and come back.

Man, aged 49 years

### Personalized Messaging

The theme *personalized messaging* refers to feedback from the biosensor about the participants’ alcohol use. Codes in this theme included alerts, real-time responses, and summary data. The majority of the participants (those with, as well as those without, previous biosensor experience) believed that receiving feedback about their alcohol use as captured by the wrist-worn alcohol biosensor would be helpful to reduce their drinking. Two main ways of receiving feedback were discussed: (1) summary data and (2) real-time alerts. Regarding summary data, individuals noted that being able to view *trends* and *patterns* in their alcohol use (eg, graphs) would be helpful to them to track their progress over time:

I think going back and looking at your data over time that could give you a sense of, you know, accomplishment that you’ve lowered your drinking over time.

Man, aged 49 years

Most of the participants (16/20, 80%) were interested in receiving alerts directly from the biosensor (eg, as a vibrating alert) as an SMS text message or from a connected app. Some of the individuals suggested the use of real-time alerts based on intoxication levels (ie, TAC levels):

I would say that you know giving me an alert and letting me know you’re drinking too much or you’ve had too many drinks, you know yeah it would help on that because, like, I was saying, a lot of times I didn’t know how many drinks I’ve had you know but, I think that if I got a reminder, or an alert saying that I’ve had enough or I’m drinking to an excess that would hopefully stop me from drinking.

Man, aged 59 years

It would be helpful if I could get notifications if I’m getting to a point where I don’t need to drink anymore, or that my alcohol level is going up too high or you know, different things like that, or what can it detect any other things in your body that is associated with the alcohol like blurred speech or stumbling or heartbeat is too high or different stuff like that.

Woman, aged 54 years

Participants also discussed wanting to receive positive feedback and encouragement about their drinking:

It could give me or let me know when I’m doing a good job with my drinking and you know, like positive messages and remind me that you know I’m doing good with my not drinking and keep up the good work, you know positive stuff....And um you know, let you know that I’m proud of you and you’re doing good, you did great today, you did great this week, you didn’t drink as much, you understand?

Woman, aged 57 years

If it could tell me, you’re doing a good job, don’t give up, I know it’s hard.

Man, aged 59 years

Several of the participants (11/20, 55%) also commented that in addition to alerts reminding them to stop or slow down their drinking, they would also like more general reminders, such as reminding them to eat when they are drinking:

Reminding me...about eating, and you know less drinking...I think that would have helped me with my stomach problems, too.

Woman, aged 53 years

### Preference for Time Wearing the Biosensor

The theme *preference for time wearing the biosensor* refers to preferences related to time spent wearing the biosensor in alignment with their drinking goals (eg, whether they wanted to quit drinking or reduce drinking). Codes in this theme included future biosensor wear time and drinking goals. Most of the participants (11/20, 55%) stated that they would want to wear the device all of the time. Many of the participants (7/11, 64%) who indicated that they would wear the sensor all of the time stated that they drank every day and would want to wear the sensor as a method to maintain their abstinence; these participants were most often those who did not typically plan their alcohol use:

You never know when that thought is gonna hit your mind: Okay, I need to stop right here and get me a drink and you stop when you go into a bar and you look down, but you don’t have the device on, you’re gonna drink ’til you’re stupid probably.

Woman, aged 54 years

Individuals who indicated that they would prefer to wear the device only some of the time agreed that they would wear the biosensor when they were drinking but did not want to wear it when they were not (6/8, 75%). These responses came from persons who did not plan to abstain from drinking; rather, they wanted to limit the amount they consumed when they did drink:

No, I wouldn’t want to wear it all the time, because I mean my alcoholism is nobody’s business and honestly, I wouldn’t mind putting it on when I was drinking though, like I would be responsible enough to say okay I’m going to drink today and I’m gonna put this on, and then I would allow it to babysit me, basically.

Man, aged 41 years

### Sharing Data With Others

The theme *sharing data with others* refers to preferences related to sharing participants’ drinking information with friends or family members or their provider. Codes in this theme included privacy, support network, and health care providers. Participants discussed how sharing the data (eg, trends in drinking and TAC levels) captured by the alcohol biosensor could help them to reduce their alcohol use. Preferences for sharing data and the purpose of sharing data varied among the participants. Participants who indicated that they would want to share the data with their friends or family members (9/20, 45%) gave reasons such as gaining additional social or emotional support:

I would like to choose who I would share with ’cause then they may call me and say hey, come on what’s going on?

Woman, aged 60 years

Most of the participants (10/20, 50%) agreed that sharing their data with a health care provider could be beneficial:

If you are sharing it with a doctor, the doctor can view it and know when....Especially if you got other ailments and other health issues and stuff like that, that would help that doctor figure out how to address your other health issues, even though you’re drinking and have a reminder, when you go see that doctor, they’re going to bring that subject up.

Woman, aged 54 years

A participant suggested sharing data directly with a therapist so that the therapist could intervene on their alcohol use in real time based on their TAC levels.

Not every participant was enthusiastic about sharing their data with friends or family members. Some of the participants (5/20, 25%) preferred to keep their data private:

You know people are judgmental so yeah, I will try to keep that to myself.

Woman, aged 56 years

To be honest, I think for now in the state I’m feeling now, I think it will just be more helpful to myself to kind of gauge, you know, where I am and know my patterns and to be able to reach out to someone should I need to reach out to someone. So, I wouldn’t want to share it, I will just want to, you know, have the information until I can kind of gauge where I am.

Man, aged 42 years

### Mental Health Support

The theme *mental health support* refers to participants’ thoughts on how mental health support could be incorporated into the biosensor. Codes in this theme included psychological aspects, motivation, and reasons for drinking. Responses in this theme spontaneously arose during the interviews (ie, direct questions about mental health support were not included in the interview guide). Drinking because of mental health conditions such as *depression* was prominent in the participants’ responses. This led to discussions regarding pairing the biosensor with a method in which they could report how they are feeling as they drink (eg, a mobile phone app). Participants indicated that understanding the relationship between their mood and drinking patterns could help them to reduce their drinking:

[T]o see if I’m drinking because I’m sad or because I’m lonely or maybe because I’m depressed ’cause then I could change some of my drinking habits.

Woman, aged 59 years

Some of the participants (5/20, 25%) shared their preference for an intervention that included a counseling or therapy component in conjunction with the biosensor:

I think you need to deal with the emotional part and the mental part of it because that’s what really...had it not been for me going through some of that therapy I went through I would still be drinking, even after going through your course [the alcohol CM study with SCRAM CAM].

Woman, aged 60 years

Two of the participants (2/20, 10%) indicated that they preferred to have the option to connect with a mental health specialist via a mobile app, should they choose to do so.

When the preliminary findings were shared during the final 4 interviews, all participants supported our interpretations.

## Discussion

### Principal Findings

In this study, we aimed to identify the desired features that would be acceptable and helpful in a wrist-worn biosensor–based alcohol intervention for adults who drink heavily. The findings could be grouped into 5 main themes: features that would influence willingness to use the biosensor, personalized messaging, preference for time wearing the biosensor, sharing data with others, and mental health support. To elaborate, the biosensor should look attractive as well as be comfortable to wear and accessible; personalized biosensor-based prompts and feedback could be helpful; for some of the participants, just wearing the biosensor could have an intervention effect; sharing data with others sounded appealing to many of the participants but not to all; and many felt that mental health support could be incorporated into the wrist-worn biosensor.

Participants were open to using the wrist-worn biosensor, indicating that these devices could be effective tools for alcohol reduction interventions in the future. As the wrist-worn Skyn biosensor is not locked onto the individual (unlike the SCRAM CAM ankle biosensor), individuals must be self-motivated to wear it. From a stages-of-change perspective [42], it is unclear whether the Skyn biosensor would be appropriate for individuals in the precontemplation stage. We only interviewed individuals who were already interested in reducing their alcohol use, which may explain the overall positive sentiment and belief that the device would be helpful. It is possible that using the biosensor may help to *open one’s eyes* about one’s alcohol use and motivate change; thus, it may complement existing effective intervention components such as motivational interviewing. Participants often discussed the importance of one’s mindset regarding intervention success, that is, one must want to change for the device to work. Examples of how the Skyn biosensor could be helpful for individuals in the contemplation stage (eg, viewing summary data to understand patterns of drinking), action stage (eg, receiving alerts in real time to slow down or stop one’s drinking), and maintenance stage (eg, wearing the device all of the time as a reminder) emerged during the interviews. Our findings suggest that people with different levels of alcohol misuse may use a biosensor differently, with some using it to try to maintain abstinence and others using it to reduce or track their drinking.

Just-in-time adaptive interventions (JITAIs) are designed to provide the right support at the right time [43]. There was much discussion about how the continuous monitoring provided by the Skyn biosensor could lead to real-time intervention, indicating that it may fit well into a JITAI [44]. In this context, individuals could help to design their own intervention features, including TAC limits at which they receive alerts and whom their data are shared with (eg, should a counselor also receive the alert?). The indication by participants that real-time feedback based on TAC alerts would be helpful is consistent with the behavior change theories that support biofeedback as an intervention tool. Additional research investigating what TAC levels should prompt an alert is warranted. A potential obstacle for using these devices in a real-time intervention is the delay of >20 minutes between alcohol intake and TAC readings [45]. In this case, certain aspects of the TAC curve—for example, the rise rate (ie, rate of intoxication) rather than peak TAC level (ie, highest level of intoxication)—may be useful to consider for an intervention. The use of mobile phone apps that include ecological momentary assessment may be especially useful in the context of a JITAI; for instance, to gauge one’s mental health and reasons for drinking [46].

Similarly, the potential to share data with health care providers could help to facilitate a patient-centered approach in which individuals act in partnership with their provider. Including the patient in their own health care decisions enhances the quality of care and has been especially useful for treating substance use and other mental health conditions [47,48]. In addition, health care providers could consider other conditions that an individual may have been diagnosed with in the context of their alcohol use to help guide recommendations and decision-making. Future research should include health care providers to understand what types of information people would want and how often they would be willing to receive such information from a biosensor.

Taken together, our findings suggest that a mobile app could be developed that houses the collected raw and interpreted data (eg, TAC levels and how mental health corresponds to daily TAC levels) in one place for the individual to view. For those who so desire, perhaps an individual’s account could be linked to observer accounts to facilitate data sharing with persons of their choosing (eg, family member and physician). It may also be helpful to have the biosensor provide prompts to connect individuals to providers who can provide alcohol-related treatment or counseling. Individuals should be able to customize the type of alerts they receive and the frequency at which they receive them. Such an app would allow for an adaptive biofeedback intervention to be implemented.

Interventions from other fields demonstrate the effectiveness of biofeedback for behavior change and can serve as examples for designing an alcohol reduction intervention that uses an alcohol biosensor; for instance, a clinical trial targeting obesity demonstrated that providing food-specific biofeedback enhanced self-efficacy and improved negative reactions to food cues and eating behaviors [28]. The literature on smoking behavior also provides evidence for the effectiveness of biofeedback on smoking cessation by reducing craving and stress [49]. Perhaps most closely related to using an alcohol biosensor to reduce drinking is the use of carbon monoxide sensors for smoking cessation, as exemplified in the studies by Marler et al [21,22]: the results from a prospective cohort of 319 adult smokers demonstrated the effectiveness of an intervention that included biofeedback via a portable carbon monoxide breath sensor, education and guidance provided through a smartphone app, and support via text-based human coaching [21,22].

Those who had previously worn the SCRAM CAM ankle biosensor were especially excited when introduced to the Skyn biosensor, likely because of the negative experiences they had with the SCRAM CAM. However, it is worth noting that most of the participants who had worn the SCRAM CAM biosensor commented on its effectiveness to help them stop drinking (in addition to the contingency of money), despite the stigma and discomfort they reported.

The Skyn biosensor is now available for both research and personal use purchase (beta testing), which brings into question how someone might use the biosensor on their own outside of a clinical trial or supervision by a health care provider. Individual users might be interested in monitoring their health in general, tracking calories, and seeking to reduce drinking. At this point, the Skyn biosensor cannot reliably estimate specific breath alcohol readings; therefore, people should not rely on the device to determine whether they can safely and legally operate a vehicle after drinking. Current availability and pricing of the Skyn biosensor may limit individual use. Regardless, individuals who choose to use the device on their own could benefit from the biofeedback provided.

### Limitations

We used a convenience sample of 20 participants, of whom 10 (50%) identified as Black women; thus, this sample should not be considered representative of all adults who drink heavily or an AUD. Despite the small sample size, however, we determined that we reached saturation by reviewing interviews on a weekly basis. Participants had to be aged at least 40 years to participate and had an average age of 55.1, SD 6.1 years; it is important to note that younger participants may have different preferences for the use of a technological tool such as the Skyn biosensor. As previously mentioned, we only interviewed persons who were currently interested in reducing their alcohol use; future research should explore whether a biosensor-based intervention would be appropriate for persons not currently interested in reducing their alcohol use. Furthermore, what people say they want in an intervention, when discussing it abstractly, may not necessarily be the key features that would result in behavior change. In addition, of the 20 participants, only 1 (5%) had ever seen or used the Skyn biosensor in real life. Because of the COVID-19 pandemic, all interviews had to be conducted remotely; therefore, we displayed an image of the biosensor and provided a verbal description over Zoom instead. As the Skyn biosensor is a new technology, and very few persons have used it, the opinions of those who have not used one yet are highly relevant. Relatedly, although conducting interviews via Zoom allowed us to include participants who lived outside of the local area during a period when much research was stalled, remote interviews come with inherent challenges. Some of these challenges include *being there differently*, in that the interviewers could not control either the setting where the individual was participating, which may result in a distracted participant, or the interruptions caused by technical issues, which may disrupt the natural flow of the interview [50]. Finally, some potential participants were unable to participate in this study because of their inability to use or access the technology (eg, no internet access).

### Conclusions

We identified 5 major themes that captured desired features of a wrist-worn biosensor–based alcohol intervention that would be acceptable and helpful to adults who drink heavily. Participants wanted a device that looked attractive and was both comfortable to wear and accessible. Participants indicated that receiving summary data about their drinking patterns and real-time alerts would be helpful. Most of the participants agreed that they would want to wear the biosensor all of the time. Some of the participants stated that they would want to share data collected from the biosensor with their friends or family members, whereas most would share their data with a health care provider. Participants also discussed receiving mental health support in conjunction with the wrist-worn biosensor. Considering these aspects, a JITAI using the wrist-worn biosensor could be developed to help adults who drink heavily reduce their alcohol use.

## Abbreviations

AUD: alcohol use disorder
CAM: continuous alcohol monitoring
CM: contingency management
JITAI: just-in-time adaptive intervention
SCRAM: Secure Continuous Remote Alcohol Monitor
TAC: transdermal alcohol concentration

## Data Availability

The data sets generated and analyzed during this study are available from the corresponding author on reasonable request.

## References

[1] (2022). Alcohol facts and statistics. National Institute on Alcohol Abuse and Alcoholism.

[2] Drinking levels defined. National Institute on Alcohol Abuse and Alcoholism.

[3] Sacks JJ, Gonzales KR, Bouchery EE, Tomedi LE, Brewer RD (2015). 2010 National and State costs of excessive alcohol consumption. Am J Prev Med.

[4] Excessive drinking is draining the U.S. economy. Centers for Disease Control and Prevention.

[5] Deaths from excessive alcohol use in the United States. Centers for Disease Control and Prevention.

[6] Mark TL, Kassed CA, Vandivort-Warren R, Levit KR, Kranzler HR (2009). Alcohol and opioid dependence medications: prescription trends, overall and by physician specialty. Drug Alcohol Depend.

[7] Hammarlund R, Crapanzano KA, Luce L, Mulligan L, Ward KM (2018). Review of the effects of self-stigma and perceived social stigma on the treatment-seeking decisions of individuals with drug- and alcohol-use disorders. Subst Abuse Rehabil.

[8] Copeland J (1997). A qualitative study of barriers to formal treatment among women who self-managed change in addictive behaviours. J Subst Abuse Treat.

[9] Grant BF (1997). Barriers to alcoholism treatment: reasons for not seeking treatment in a general population sample. J Stud Alcohol.

[10] Ali MM, Teich JL, Mutter R (2017). Reasons for not seeking substance use disorder treatment: variations by health insurance coverage. J Behav Health Serv Res.

[11] Hester RK, Miller JH (2006). Computer-based tools for diagnosis and treatment of alcohol problems. Alcohol Res Health.

[12] Sinadinovic K, Berman AH, Hasson D, Wennberg P (2010). Internet-based assessment and self-monitoring of problematic alcohol and drug use. Addict Behav.

[13] Staiger PK, O'Donnell R, Liknaitzky P, Bush R, Milward J (2020). Mobile apps to reduce tobacco, alcohol, and illicit drug use: systematic review of the first decade. J Med Internet Res.

[14] Colbert S, Thornton L, Richmond R (2020). Smartphone apps for managing alcohol consumption: a literature review. Addict Sci Clin Pract.

[15] Swift R (2003). Direct measurement of alcohol and its metabolites. Addiction.

[16] Noorbergen TJ, Adam MT, Attia JR, Cornforth DJ, Minichiello M (2019). Exploring the design of mHealth systems for health behavior change using mobile biosensors. Commun Assoc Inform Syst.

[17] Michie S, Abraham C (2004). Interventions to change health behaviours: evidence-based or evidence-inspired?. Psychol Health.

[18] Carver CS, Scheier MF (1982). Control theory: a useful conceptual framework for personality-social, clinical, and health psychology. Psychol Bull.

[19] Bandura A (1998). Health promotion from the perspective of social cognitive theory. Psychol Health.

[20] Papathanasiou G, Georgakopoulos D, Papageorgiou E, Zerva E, Michalis L, Kalfakakou V, Evangelou A (2013). Effects of smoking on heart rate at rest and during exercise, and on heart rate recovery, in young adults. Hellenic J Cardiol.

[21] Marler JD, Fujii CA, Utley DS, Tesfamariam LJ, Galanko JA, Patrick H (2019). Initial assessment of a comprehensive digital smoking cessation program that incorporates a mobile app, breath sensor, and coaching: cohort study. JMIR Mhealth Uhealth.

[22] Marler JD, Fujii CA, Galanko JA, Balbierz DJ, Utley DS (2021). Durability of abstinence after completing a comprehensive digital smoking cessation program incorporating a mobile app, breath sensor, and coaching: cohort study. J Med Internet Res.

[23] Beard E, West R (2012). Pilot study of the use of personal carbon monoxide monitoring to achieve radical smoking reduction. J Smok Cessat.

[24] Choi W, Kim C, Lee O (2013). Effects of brief smoking cessation education with expiratory carbon monoxide measurement on level of motivation to quit smoking. Korean J Fam Med.

[25] Risser NL, Belcher DW (1990). Adding spirometry, carbon monoxide, and pulmonary symptom results to smoking cessation counseling: a randomized trial. J Gen Intern Med.

[26] Jamrozik K, Vessey M, Fowler G, Wald N, Parker G, Van Vunakis H (1984). Controlled trial of three different antismoking interventions in general practice. Br Med J (Clin Res Ed).

[27] Imperatori C, Mancini M, Della Marca G, Valenti EM, Farina B (2018). Feedback-based treatments for eating disorders and related symptoms: a systematic review of the literature. Nutrients.

[28] Teufel M, Stephan K, Kowalski A, Käsberger S, Enck P, Zipfel S, Giel KE (2013). Impact of biofeedback on self-efficacy and stress reduction in obesity: a randomized controlled pilot study. Appl Psychophysiol Biofeedback.

[29] Wang Y, Fridberg DJ, Leeman RF, Cook RL, Porges EC (2019). Wrist-worn alcohol biosensors: strengths, limitations, and future directions. Alcohol.

[30] Greenfield TK, Kerr WC (2008). Alcohol measurement methodology in epidemiology: recent advances and opportunities. Addiction.

[31] Wang Y, Fridberg DJ, Shortell DD, Leeman RF, Barnett NP, Cook RL, Porges EC (2021). Wrist-worn alcohol biosensors: applications and usability in behavioral research. Alcohol.

[32] Kilmer B, Nicosia N, Heaton P, Midgette G (2013). Efficacy of frequent monitoring with swift, certain, and modest sanctions for violations: insights from South Dakota's 24/7 Sobriety Project. Am J Public Health.

[33] Villalba K, Cook C, Dévieux JG, Ibanez GE, Oghogho E, Neira C, Cook RL (2020). Facilitators and barriers to a contingency management alcohol intervention involving a transdermal alcohol sensor. Heliyon.

[34] Boyce C, Neale P (2006). Conducting in-depth interview: a guide for designing and conducting in-depth interviews for evaluation input. Pathfinder International Tool Series, Monitoring and Evaluation-2.

[35] Yardley L, Bradbury K, Morrison L (2021). Using qualitative research for intervention development and evaluation. Qualitative Research in Psychology: Expanding Perspectives in Methodology and Design.

[36] Glaser B, Strauss A (1967). Discovery of Grounded Theory: Strategies for Qualitative Research.

[37] Archibald MM, Ambagtsheer RC, Casey MG, Lawless M (2019). Using zoom videoconferencing for qualitative data collection: perceptions and experiences of researchers and participants. Int J Qual Method.

[38] Braun V, Clarke V (2006). Using thematic analysis in psychology. Qual Res Psychol.

[39] Elliott V (2018). Thinking about the coding process in qualitative data analysis. Qual Report.

[40] Software solutions for leading researchers and educators. QSR International.

[41] Lincoln Y, Guba E (1985). Naturalistic Inquiry.

[42] DiClemente CC, Hughes SO (1990). Stages of change profiles in outpatient alcoholism treatment. J Subst Abuse.

[43] Nahum-Shani I, Smith SN, Spring BJ, Collins LM, Witkiewitz K, Tewari A, Murphy SA (2018). Just-in-time adaptive interventions (JITAIs) in mobile health: key components and design principles for ongoing health behavior support. Ann Behav Med.

[44] Fridberg DJ, Wang Y, Porges E (2022). Examining features of transdermal alcohol biosensor readings: a promising approach with implications for research and intervention. Alcohol Clin Exp Res.

[45] Fairbairn CE, Kang D (2019). Temporal dynamics of transdermal alcohol concentration measured via new-generation wrist-worn biosensor. Alcohol Clin Exp Res.

[46] Wray TB, Monti PM, Kahler CW, Guigayoma JP (2020). Using ecological momentary assessment (EMA) to explore mechanisms of alcohol-involved HIV risk behavior among men who have sex with men (MSM). Addiction.

[47] Institute of Medicine, Committee on Crossing the Quality Chasm: Adaptation to Mental Health and Addictive Disorders, Board on Health Care Services (2006). Improving the Quality of Health Care for Mental and Substance-Use Conditions.

[48] Bradley KA, Kivlahan DR (2014). Bringing patient-centered care to patients with alcohol use disorders. JAMA.

[49] Keilani M, Steiner M, Crevenna R (2022). The effect of biofeedback on smoking cessation-a systematic short review. Wien Klin Wochenschr.

[50] Oliffe JL, Kelly MT, Gonzalez Montaner G, Yu Ko WF (2021). Zoom interviews: benefits and concessions. Int J Qual Method.

